# Two-Dimensional Metal Organic Framework Nanosheets as Bifunctional Catalyst for Electrochemical and Photoelectrochemical Water Oxidation

**DOI:** 10.3389/fchem.2020.604239

**Published:** 2020-11-04

**Authors:** Chang Liu, Xiaochen Shen, Grayson Johnson, Yulu Zhang, Changlin Zhang, Jiafu Chen, Lingyan Li, Colton Sheehan, Zhenmeng Peng, Sen Zhang

**Affiliations:** ^1^Department of Chemistry, University of Virginia, Charlottesville, VA, United States; ^2^Department of Chemical and Biomolecular Engineering, University of Akron, Akron, OH, United States

**Keywords:** metal organic framework, nanosheet, photoelectrochemical water oxidation, oxygen evolution reaction, bifunctional catalyst

## Abstract

Electrochemical (EC) and photoelectrochemical (PEC) water splitting represent promising strategies for renewable energy conversion and fuel production and require design of efficient catalysts for the oxygen evolution reaction (OER). Herein, we report the synthesis of two-dimensional (2D) Co-based metal organic framework (Co-MOF) nanosheets and their bifunctional catalytic properties for both EC and PEC OER. Benefiting from the large surface area and abundant isolated metal active sites, the Co-MOF nanosheets exhibited excellent OER activity and stability. The efficient electron–hole generation and separation of the nanosheets, owing to dimensional confinement, contributed to an improved visible light response in PEC OER. This study presents a new strategy to design EC/PEC bifunctional catalyst utilizing unique structural and electronic features of 2D MOF.

## Introduction

Electrochemical (EC) and photoelectrochemical (PEC) water splitting are recognized as two attractive processes for clean fuel hydrogen (H_2_) generation via the conversion of renewable electrical energy into chemical energy and have aroused intense research interest in recent years (Fujishima and Honda, 1972; Zhong and Gamelin, 2010; Kim and Choi, 2014; Zhang et al., 2020). Nevertheless, the oxidative half-cell reaction at the anode, oxygen evolution reaction (OER), generally requires high overpotential as driving force owing to the intrinsically sluggish kinetics. To this end, electrocatalysts and photoelectrocatalysts have been extensively pursued to promote the reaction kinetics and improve the energy conversion efficiency. For practical purpose, efficient catalysts that work for both EC and PEC OER are required to allow the seamless operation of water splitting in the presence and absence of sunlight, maximizing the process efficiency. Such bifunctional catalyst development mainly resides in metal oxide nanomaterials, such as bismuth vanadate (BiVO_4_) (Kim and Choi, 2014), hematite (α-Fe_2_O_3_) (Zhong and Gamelin, 2010), and tungsten trioxide (WO_3_) (Sarnowska et al., 2016), most of which, however, still cannot achieve desirable properties for the two processes simultaneously.

Metal organic frameworks (MOFs) are a crystalline porous material with the coordinations of transitional metal nodes and organic linkers, which have attracted significant interest for applications in gas sorption, energy storage, and heterogeneous catalysis (Rodenas et al., 2015; Dhakshinamoorthy et al., 2016; Sheberla et al., 2017). A great variety of two-dimensional (2D) MOFs were also discovered with emergent physiochemical properties owing to the 2D confinement (Peng et al., 2014; Rodenas et al., 2015; Zhan and Zeng, 2016). In particular, 2D MOFs possess extremely large specific surface area and a high concentration of isolated metal ion sites on the surface, making them unique materials for the studies of heterogeneous catalysis (Li et al., 2011). Meanwhile, benefiting from an excess electron density of organic linkers, MOFs are endowed with semiconductor-like properties in which photons can be absorbed and transferred through organic linkers to bound metal ions, an important characteristic in photoelectrocatalysis (Dhakshinamoorthy et al., 2016; Zhang et al., 2016). Therefore, despite the lack of previous experimental report, 2D MOFs could potentially provide a catalytic structure that allows the dual-functionality in both EC and PEC OER catalysis.

Herein, we report, for the first time, the synthesis of 2D cobalt-based MOF (Co-MOF) nanosheets and their catalytic properties for EC and PEC OER. The Co-MOF nanosheets were obtained through the exfoliation treatment of premade MOF-71 bulk materials, with consecutive processes of hydrothermal reaction in water and sonication in isopropanol. The resultant Co-MOF nanosheets demonstrated high activity for EC OER and improved visible light response for PEC OER. The enhanced EC and PEC catalytic performances of 2D Co-MOF nanosheets, comparing to bulk MOF-71, is associated with the large surface area, abundant catalytic sites, and low-dimension confinement effect of 2D structure, suggesting the high potential of modulating 2D MOF-based catalysts for EC and PEC water electrolyzer or other energy/fuel production devices.

## Experimental Section

### Materials

All chemicals were used as received without further purification. Cobalt nitrate hexahydrate [Co(NO_3_)_2_.6H_2_O, 98%], terephthalic acid (1,4-BDC, 98%), and dimethylformamide (DMF, 99.8%) were purchased from Millipore Sigma. Isopropanol (2-propanol, 99.9%) was purchased from Fisher Scientific.

### Synthesis of Bulk MOF-71

Bulk MOF-71 was synthesized using the reported method (Rosi et al., 2005). In a typical synthesis, 389 mg of Co(NO_3_)_2_ ·6H_2_O, 74 mg of 1,4-BDC, and 20 ml of DMF were mixed together and transferred into an autoclave. The hydrothermal reaction in the autoclave took place at 110°C for 12 h. The resultant product was collected by centrifugation at 6,000 rpm for 5 min, and the powder was washed twice consecutively with DMF solvent for the removal of unreacted organic linker. Finally, the material was dried in an oven at 70°C overnight, in which it is stored and used in the subsequent experiment.

### Synthesis of Co-MOF Nanosheets

Twenty milligrams of as-synthesized bulk MOF-71 and 20 ml of deionized water were mixed and transferred into an autoclave. The autoclave was maintained at 90°C for 12 h. The dispersion was then sonicated for 6 h and aged for 12 h. The sediment was collected and mixed with 10 ml of isopropanol. The mixture was then sonicated again for 6 h. Finally, the Co-MOF nanosheets were obtained by collecting the colloid dispersion with precipitation removal and evaporating the solvent at room temperature. The yield of Co-MOF nanosheets was 20% (4 mg of Co-MOF nanosheets can be obtained from 20 mg of bulk MOF-71).

### Synthesis of Co(OH)_2_ Nanosheets

Ten milliliters of 0.2 M Co(NO_3_)_2_ aqueous solution was purged under argon for the removal of oxygen, to which 2 ml of 1 M NaOH aqueous solution was dropwise added. The obtained dispersion was aged for 15 min. The product was then washed three times by adding deionized water and centrifuged at 6,000 rpm for 5 min. The material was dried at room temperature.

## Characterization

The transmission electron microscopy (TEM) images of the prepared samples were obtained using a JEOL JEM-1230 microscope (acceleration voltage, 120 kV). High-resolution TEM (HRTEM) images were taken on a FEI Tecnai G2 F20 at 200 kV. X-ray diffraction (XRD) patterns were collected on a Bruker AXS Dimension D8 X-Ray diffractometer with a Cu Kα radiation source. The UV–Vis absorption spectra were recorded using an HP 8453 spectrometer. Fourier transform infrared (FTIR) spectroscopy experiments were conducted on a Thermo Scientific Nicolet 6700 spectrometer.

### Rotating Disk Electrode Thin Film Preparation

Catalyst films were formed on a glassy carbon (GC) rotating disk electrode (RDE) (5 mm diameter) for electrochemical measurements. The catalyst ink was prepared by dispersing 2 mg of catalyst powders [bulk MOF-71, Co-MOF nanosheets or Co(OH)_2_ nanosheets] in isopropanol (990 μl) and Nafion ionomer (10 μl) (V_isopropanol_/V_nafion_ = 99:1) and sonicating for 30 min. The GC-RDE was typically cleaned by sonication and rinsing in deionized water and polishing with alumina nanopowders for 2 min. The prepared catalyst ink (5 μl) was spin casted twice on the GC-RDE at 350 rpm, which contained 10 μg of catalyst.

### Electrochemical Measurements

The electrochemical property of catalyst was evaluated using a CHI 760D potentiostat (CH Instrument, Inc.) and a rotating disk electrode controller (AFMSRCE, Pine Instrument Co.). A three-electrode system, consisting of a glassy carbon working electrode, platinum wire counter electrode, and Ag/AgCl reference electrode (CHI 111), was used in the study. The electrolyte was 1 M potassium hydroxide (KOH) aqueous solution. The Ag/AgCl reference electrode was calibrated vs. reversible hydrogen electrode (RHE) potential by measuring the Pt electrode in hydrogen atmosphere. The OER overpotential (η) were calculated using η = E (vs. RHE) – 1.23 V. All potentials are reported vs. RHE. Linear sweep voltammetry (LSV) was collected in the potential window of 1.03–1.63 V at a scan rate of 5 mV s^−1^. Electrochemical impedance spectroscopy (EIS) was measured at initial potential of 1.58 V from 1 to 105 Hz. Current–time (I–t) curve was collected at a potential of 1.58 V. For the photoelectrocatalytic property investigation, a Xeon solar simulator (PerfectLight, China) was used as a light source, with an AM 1.5 G filter and a power density at the RDE surface adjusted to 100 mW cm^−2^ before testing. The RDE would rotate at speed of 1,600 rpm during our measurement.

## Results and Discussion

**Figure 1:**
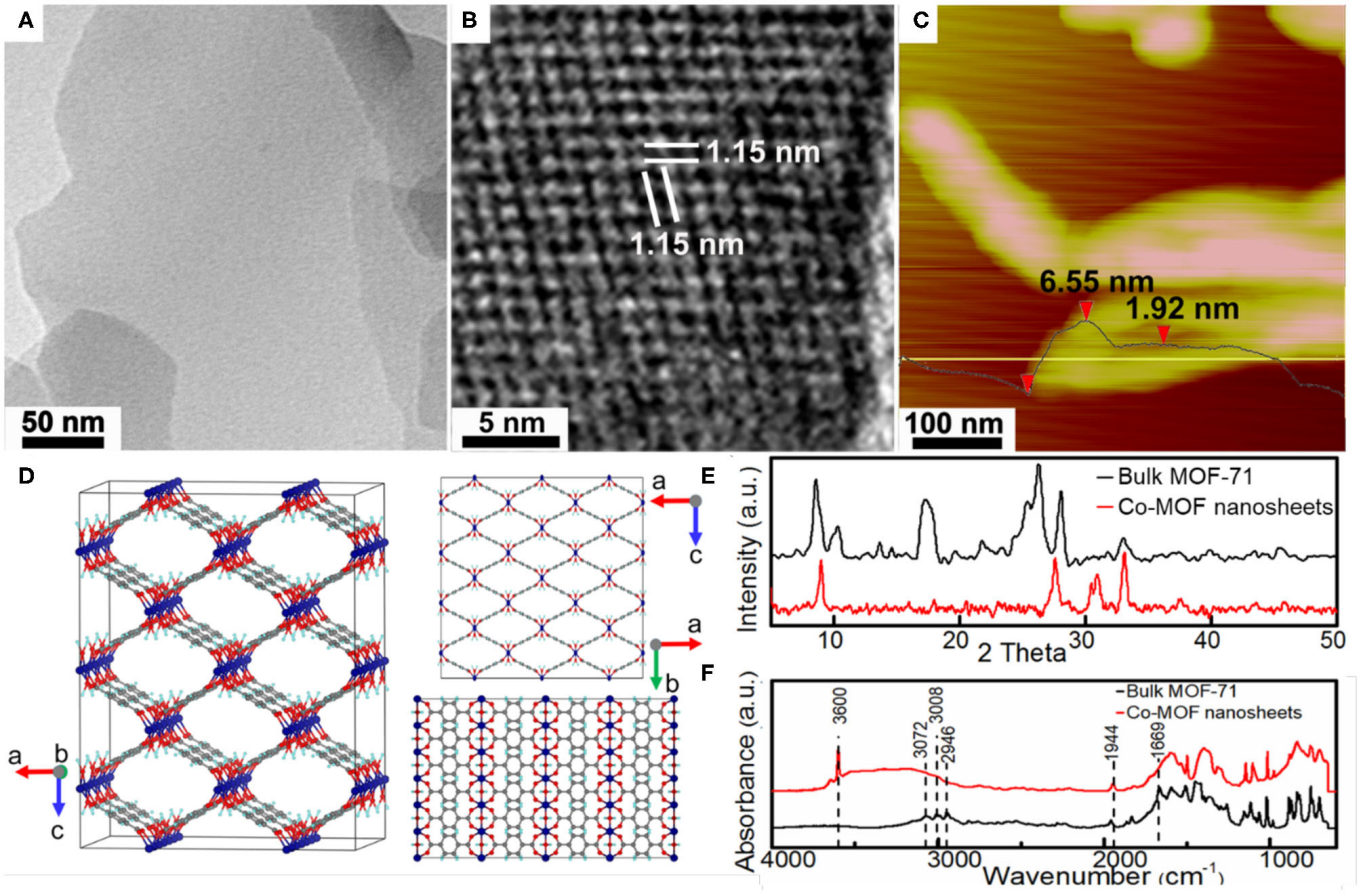
Structural characterizations of the as-synthesized Co-based metal organic framework (Co-MOF) nanosheets: **(A)** transmission electron microscopy (TEM) image, **(B)** high-resolution TEM (HRTEM) image, **(C)** atomic force microscopy (AFM) image, and **(D)** crystalline structure of bulk MOF-71 (Co, C, O, and H atoms are shown in purple, gray, red, and blue) along different crystallographic axes; **(E)** X-ray diffraction (XRD) and **(F)** Fourier transform infrared (FTIR) of bulk MOF-71 and Co-MOF nanosheets.

Figure 1A shows the TEM image of as-synthesized Co-MOF nanosheets. The uniform and low contrast of individual nanosheet indicates its thinness. The observation of the Tyndall effect from a colloid suspension of the nanosheets was consistent with their nanoscale characteristics (Supplementary Figure 1). In comparison, the obtained bulk MOF-71 was comprised of closely stacking layers (Supplementary Figure 2), which was verified by TEM characterizations and suggested the exfoliation mechanism for the nanosheet formation. Careful HRTEM characterizations confirmed microporous characteristic of the Co-MOF nanosheets, with an average molecular channel spacing of 1.07 nm (Figure 1B). The nanosheet thickness was limited to a few nanometers based on atomic force microscopy (AFM) measurements (Figure 1C), in consistence with the TEM results. The XRD pattern indicated a crystalline structure of the as-synthesized bulk MOF-71 and was in agreement with that reported for MOF-71, which consists of Co^II^ square-pyramidal coordination geometry networks (Figures 1D,E) (Li et al., 2015; Chisca et al., 2016). In comparison, the XRD pattern of synthesized Co-MOF nanosheets shows fewer reflection peaks, which could be associated with favorable orientation along the basal plane (Figure 1E). The DMF ligand substitution with water during exfoliation could also introduce the new diffraction peaks at a large diffraction angle. Figure 1F shows the FTIR results of the Co-MOF nanosheets and the bulk MOF-71. The peaks at 3,072, 3,008, and 2,946 cm^−1^ were assigned to stretching vibration of =C–H in aromatic ring and –CH and –CH_3_ stretching vibrations in DMF, respectively (Colthup et al., 1990). The band at 1,944 cm^−1^ was attributed to the aromatic carbon bond, corresponding to the spectrum of 1,4-BDC and confirming the ligands in the coordination network (Supplementary Figure 3) (Colthup et al., 1990). The bulk MOF-71 exhibited one absorbance band at 1,669 cm^−1^, which was ascribed to the carboxyl group in DMF molecules (Colthup et al., 1990). The band at 1,669 cm^−1^ disappeared on the FTIR of the Co-MOF nanosheets. Instead, a new peak at 3,600 cm^−1^ together with a broad bump between 3,600 and 3,000 cm^−1^, characteristics of hydroxyl groups, appeared after the bulk MOF-71 was exfoliated into nanosheets. These changes indicated that the DMF ligands were substituted with water molecules after the exfoliation process.

**Figure 2:**
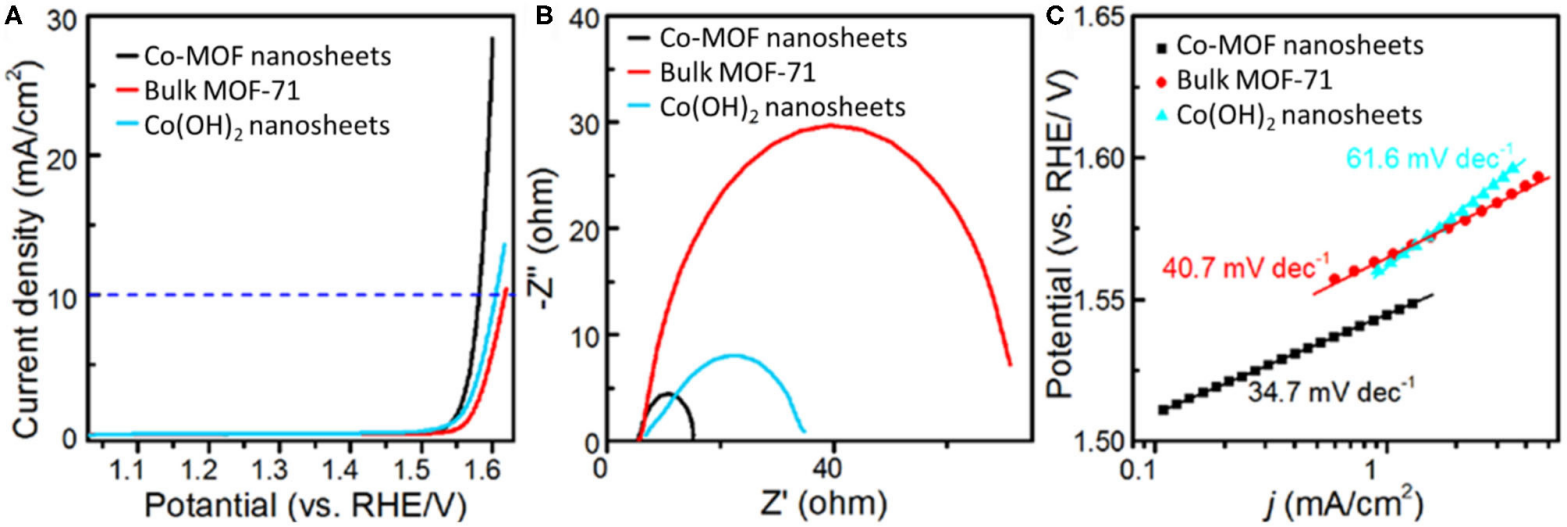
Electrochemical measurements in 1 M KOH electrolyte: **(A)** iR-corrected linear sweep voltammetry (LSV), **(B)** Nyquist plots, and **(C)** Tafel plots of Co-MOF nanosheets, bulk MOF-71, and Co(OH)_2_ nanosheets.

The catalytic properties of Co-MOF nanosheets for EC OER were investigated in 1 M KOH electrolyte. The LSV, with an onset potential at about 1.48 V and an exponential increase in current afterward, confirmed that the Co-MOF nanosheets were highly active for the OER (Figure 2A). The η required to achieve a current density of 10 mA cm^−2^ was determined to be 350 mV (Supplementary Figure 4) (McCrory et al., 2015). In comparison, 380 and 370 mV of η were needed for the bulk MOF-71 and the Co(OH)_2_ nanosheets, respectively (Supplementary Figures 5–7), demonstrating an improved catalytic activity of the Co-MOF nanosheets. Supplementary Table 1 summarizes EC OER performances of recently reported 2D MOF and MOF-derived materials, which also confirms the high activity of our Co-MOF nanosheets. Previous studies related to Co-based catalysts reported that high-valance state cobalt is responsible for OER electrocatalysis (Burke et al., 2015). A small oxidation peak prior to water oxidation was observed on the LSV of the Co-MOF nanosheets, which may be attributed to the transformation of surface Co^II^ to Co^III^. The oxidation peak of the Co-MOF nanosheets was centered at around 1.17 V, significantly lower than that of the bulk MOF-71 (1.28 V) and the Co(OH)_2_ nanosheets (1.30 V). This result suggested more efficient generation of Co^III^ sites on the 2D Co-MOF nanosheets and could partially account for their higher OER activity (Supplementary Figures 8–10). In addition, the larger electrochemical surface area of the Co-MOF nanosheets was beneficial to expose more active sites, thereby allowing for a higher activity than the bulk MOF-71. The larger electrochemical surface area was corroborated with an about 12-time increase in the double layer capacitance of the MOF nanosheets compared to the bulk MOF (Supplementary Figure 11). The Nyquist plots in Figure 2B show that the Co-MOF nanosheets possessed the smallest interfacial charge transfer resistance compared with the bulk MOF-71 and the Co(OH)_2_ nanosheets, confirming the promoted OER kinetics. The Tafel slope is another important index for OER kinetic evaluation and a description for the relationship between electrode potential and OER current density. The Tafel data of the Co-MOF nanosheets, the bulk MOF-71, and the Co(OH)_2_ nanosheets are shown in Figure 2C. The Tafel slopes of these catalysts were in the same range as that of the well-studied IrO_2_ (49 mV dec^−1^) and Ir/C (40 mV dec^−1^) OER catalysts (Trotochaud et al., 2012; Gong et al., 2013). The Tafel slope of the Co-MOF nanosheets was measured to be 34.7 mV dec^−1^, smaller than the other two samples [40.7 mV dec^−1^ for bulk MOF and 61.6 mV dec^−1^ for Co(OH)_2_], which could suggest the varied OER mechanisms on their surfaces (Gong et al., 2013).

**Figure 3:**
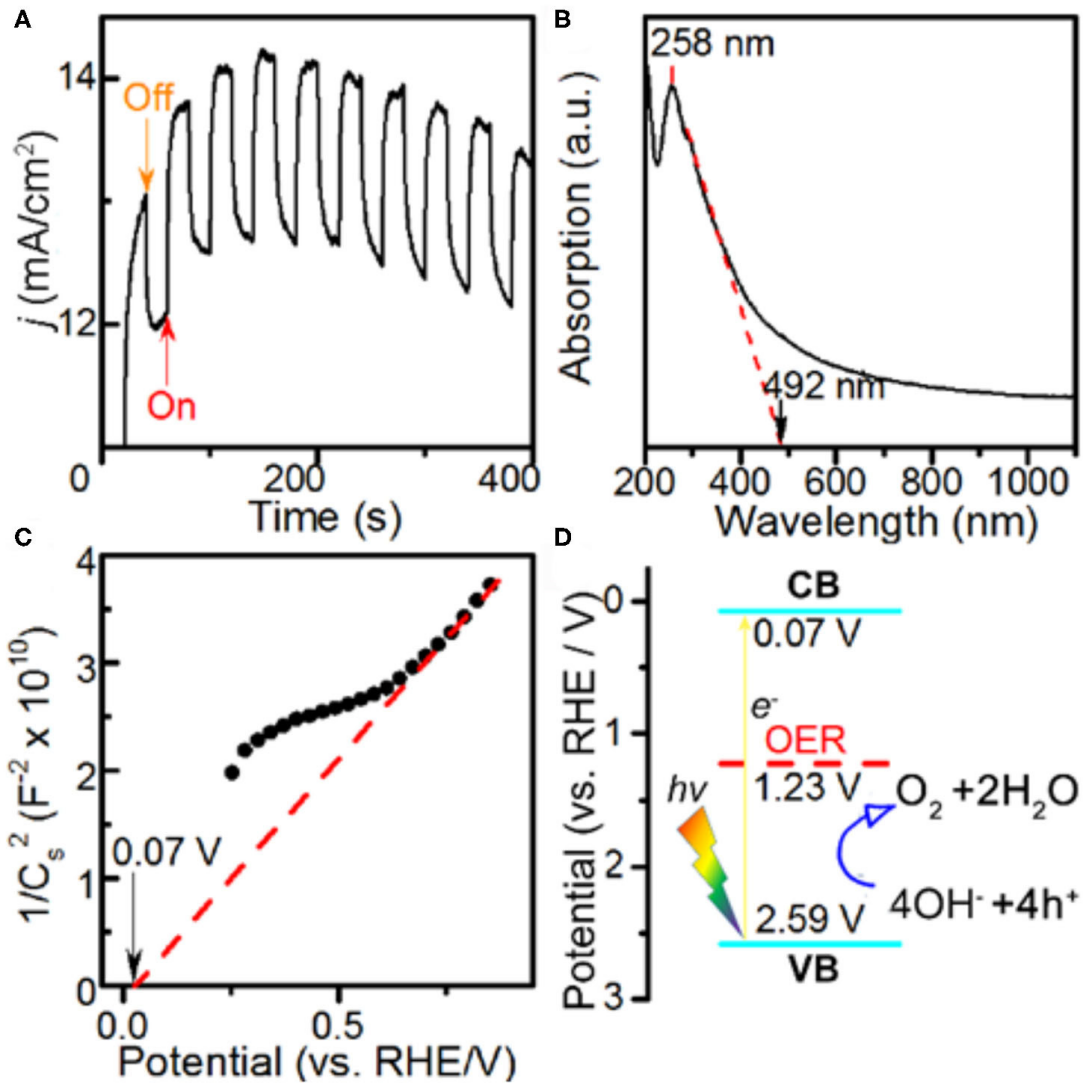
Photoelectrochemical measurements in 1 M KOH electrolyte and characterizations: **(A)** potentiostatic current response at E = 1.582 V vs. reversible hydrogen electrode (RHE) with and without light illumination, **(B)** UV–Vis spectrum, **(C)** Mott–Schottky plot, and **(D)** band alignment of the Co-MOF nanosheets.

The PEC properties of the Co-MOF nanosheets were investigated in the same electrolyte. The PEC current profile was collected under AM 1.5 G visible light illumination at intervals of 20 s (Figure 3A). The immediate changes in the current density with on and off switches of the light demonstrated a significant photo response of the Co-MOF nanosheets (Supplementary Figure 12), suggesting that they are active for PEC OER. Under the specific testing condition, the OER current density increased by about 1.37 mA cm^−2^ under illumination. The photo response was larger than that using the bulk MOF-71 (1.23 mA cm^−2^, Supplementary Figure 13). The increased PEC response of the Co-MOF nanosheets could be attributed to more efficient charge separation in 2D structure (O'Regan and Grätzel, 1990; King et al., 2013). The Co-MOF nanosheets exhibited a mixed mode of absorbance, evidenced by the UV–Vis spectrum (Figure 3B). The peak at 258 nm could be assigned to the π-π transition in the conjugated ligand (Sarnowska et al., 2016). Besides, a small shoulder peak at around 300 nm was observed together with continuous absorption of light in a broad wavelength range, which was more like a semiconductor. The band gap was determined to be 2.52 V based on the UV–Vis measurements. The flat band potential of the Co-MOF nanosheets was measured using the Mott–Schottky method (Figure 3C) (Gryse et al., 1975). The positive slope indicated that the Co-MOF nanosheets were an n-type semiconductor (Lin et al., 2014). The energy level of the conduction band (CB) bottom was determined to be 0.07 V vs. RHE. Based on the UV–Vis absorbance data and the Mott–Schottky plot, we could determine the energy level of the valence band (VB) top (2.59 V vs. RHE) and draw the energy level scheme (Figure 3D). The band alignment of bulk MOF-71 is shown in Supplementary Figure 14, which indicated a smaller band gap than Co-MOF nanosheets. Under the illumination of visible light, there would be excitation of electrons from the VB to CB band. Thus, the holes generated in the VB band possess a more positive potential than the Nernstian potential of 1.23 V for OER, which creates an additional driving force for the reaction and demonstrates that the PEC process is thermodynamically feasible on the Co-MOF nanosheets (Linic et al., 2015).

**Figure 4:**
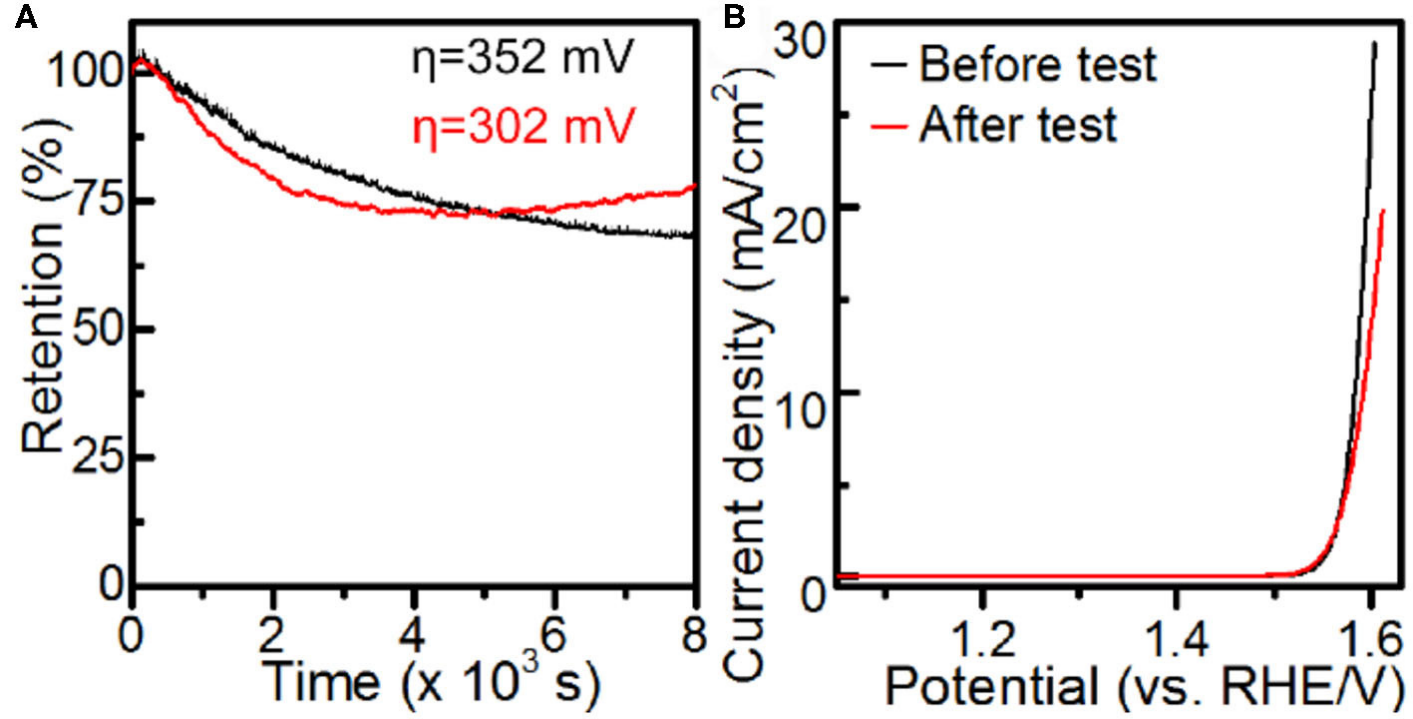
Electrochemical measurements in 1 M KOH: **(A)** chronoamperometry and **(B)** iR-corrected LSV using Co-MOF nanosheets.

The catalytic durability of the Co-MOF nanosheets was evaluated by conducting chronoamperometry (CA) experiments at an η of 352 mV. The Co-MOF nanosheets exhibited a promising durability, with a gradual decrease in the current and 68% of the activity retained after 8,000 s of CA test (Figure 4 and Supplementary Figure 15). The deactivation of the catalyst could be associated with oxidation and aggregation of surface active Co sites caused by the high overpotential, evidenced by an increase in the charge transfer resistance and formation of small nanoparticles on the tested Co-MOF nanosheets (Supplementary Figures 16, 17). The conclusion was supported by the durability experiments conducted at a lower overpotential of 302 mV, where the tested Co-MOF nanosheets retained 80% of the initial activity and showed significantly less generation of particles (Supplementary Figures 17–19). Moreover, the photo response was retained for the electrode after durability test (Supplementary Figure 20), which indicated an encouraging PEC stability of the Co-MOF nanosheets.

## Conclusions

In summary, we synthesized Co-MOF nanosheets and studied their catalytic properties in EC and PEC OER processes. Characterizations showed that these Co-MOF nanosheets possessed the MOF-71 structure, with lattice DMF ligands substituted with water molecules in comparison with the bulk MOF-71. The Co-MOF nanosheets were found with interesting properties for both EC and PEC OER, demonstrating the potential use of 2D MOF structures as the bifunctional catalysts for water electrolyzers. The Co-MOF nanosheet catalyst exhibited a clear improvement in OER activity relative to the bulk MOF-71 (350 vs. 380 mV of η at 10 mA cm^−2^ current density), which was attributed to a significantly larger surface area and more efficient generation of high valence state Co active sites. The more intense response to visible light under the PEC condition comparing to its bulk counterpart was associated with more efficient charge separation in 2D structure. This work presents the first example of 2D MOF nanosheets for EC and PEC OER and suggests that the further tuning of 2D MOF nanostructures may lead to new opportunities in the design and construction of efficient catalysts for EC and PEC combined-mode water electrolyzers.

## Data Availability Statement

All datasets generated for this study are included in the article/Supplementary Material.

## Author Contributions

The project was conceived by CL under the supervision of ZP and SZ. Catalyst synthesis, structural characterization, and catalysis measurement were performed by CL, XS, GJ, YZ, and CS. The analysis and interpretation of all spectra were done by CL, CZ, and JC. The AFM image was taken by CL and LL. All authors contributed to the article and approved the submitted version.

## Conflict of Interest

The authors declare that the research was conducted in the absence of any commercial or financial relationships that could be construed as a potential conflict of interest.
