# Supplementary material for: Two-Dimensional Metal Organic Framework Nanosheets as Bifunctional Catalyst for Electrochemical and Photoelectrochemical Water Oxidation
Source: Front Chem. 2020 Nov 4;8:604239. doi: 10.3389/fchem.2020.604239 (PMC7672199; doi:10.3389/fchem.2020.604239)
Supplement: Supplementary file 1 [file Data_Sheet_1.docx]

Supplementary Material for

**Two-Dimensional Metal-Organic Framework Nanosheets as Bifunctional Catalyst for Electrochemical and Photoelectrochemical Water Oxidation**

Chang Liu^1, #^, Xiaochen Shen^2, #^, Grayson Johnson^1^, Yulu Zhang^1^, Changlin Zhang^2^, Jiafu Chen^2^, Lingyan Li^2^, Colton Sheehan^1^, Zhenmeng Peng^2, *^, Sen Zhang^1, *^

^1^Department of Chemistry, University of Virginia, Charlottesville, Virginia 22904, United States

^2^Department of Chemical and Biomolecular Engineering, University of Akron, Akron, Ohio, 44325, United States.

*** Correspondence:**Zhenmeng Peng, Sen Zhang

[zpeng@uakron.edu](mailto:zpeng@uakron.edu), [sz3t@virginia.edu](mailto:sz3t@virginia.edu)

# These authors contributed equally to this work.

**
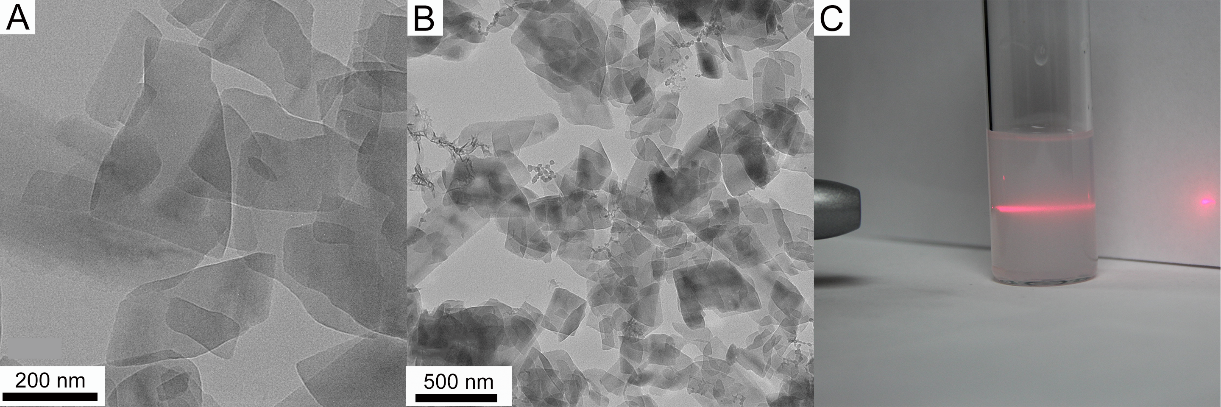
**

**Figure S1 (A, B)** TEM images of Co-MOF nanosheets, and **(C)** digital photograph of the Tyndall effect for Co-MOF nanosheets colloid suspension in isopropanol.

**
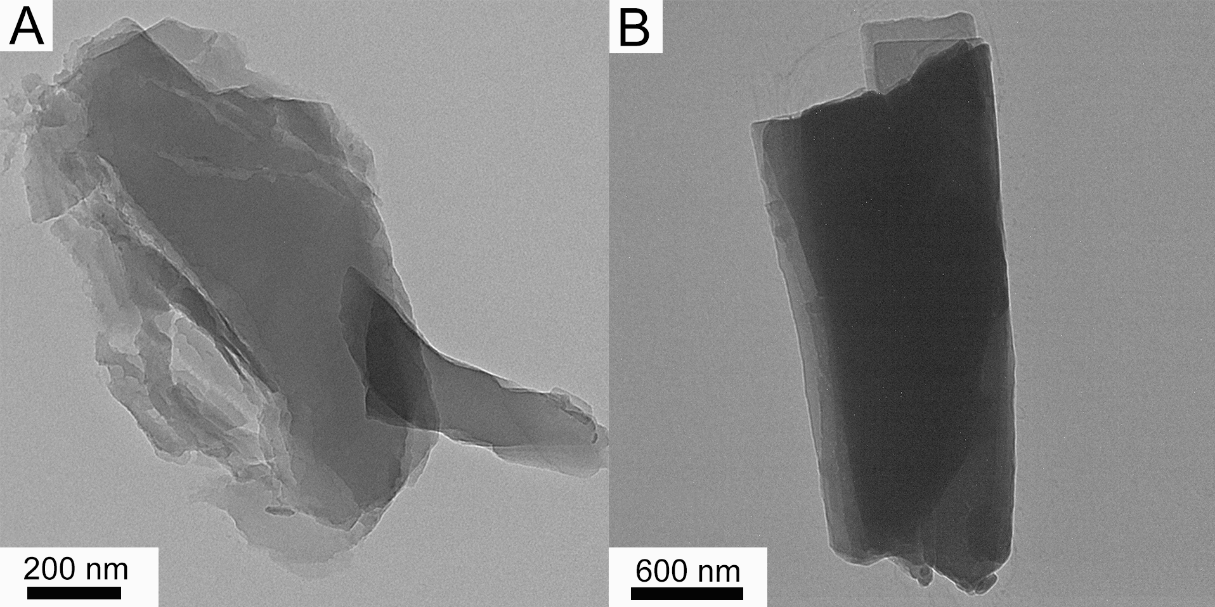
**

**Figure S2** TEM images of bulk MOF-71.

**
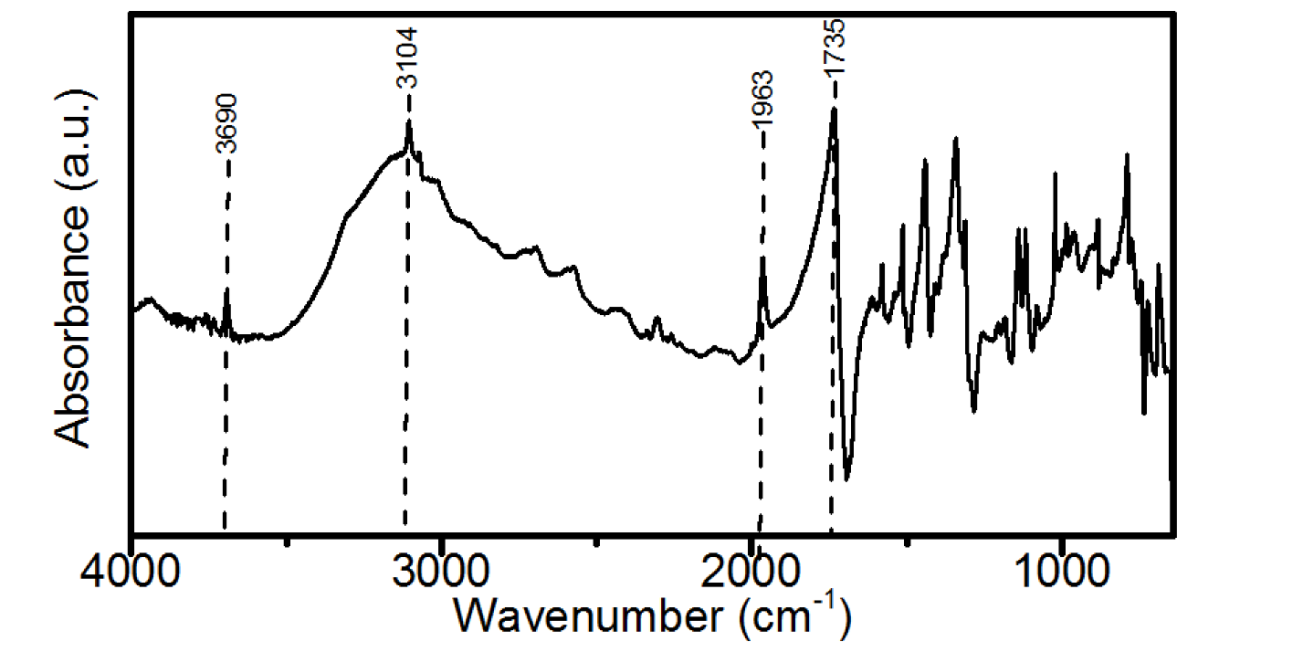
**

**Figure S3** FTIR spectrum of terephthalic acid powder.


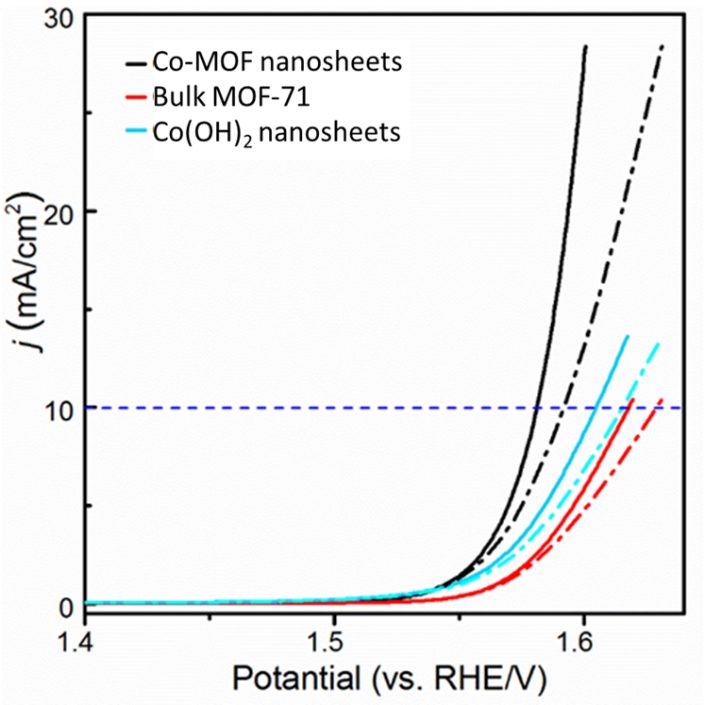


**Figure S4** Electrochemical measurements of Co-MOF nanosheets, bulk MOF-71 and Co(OH)_2_ nanosheets, with (solid line) and without (dashed line) iR-correction.

**

**

**Figure S5** TEM images of the prepared Co(OH)_2_ nanosheets.

**
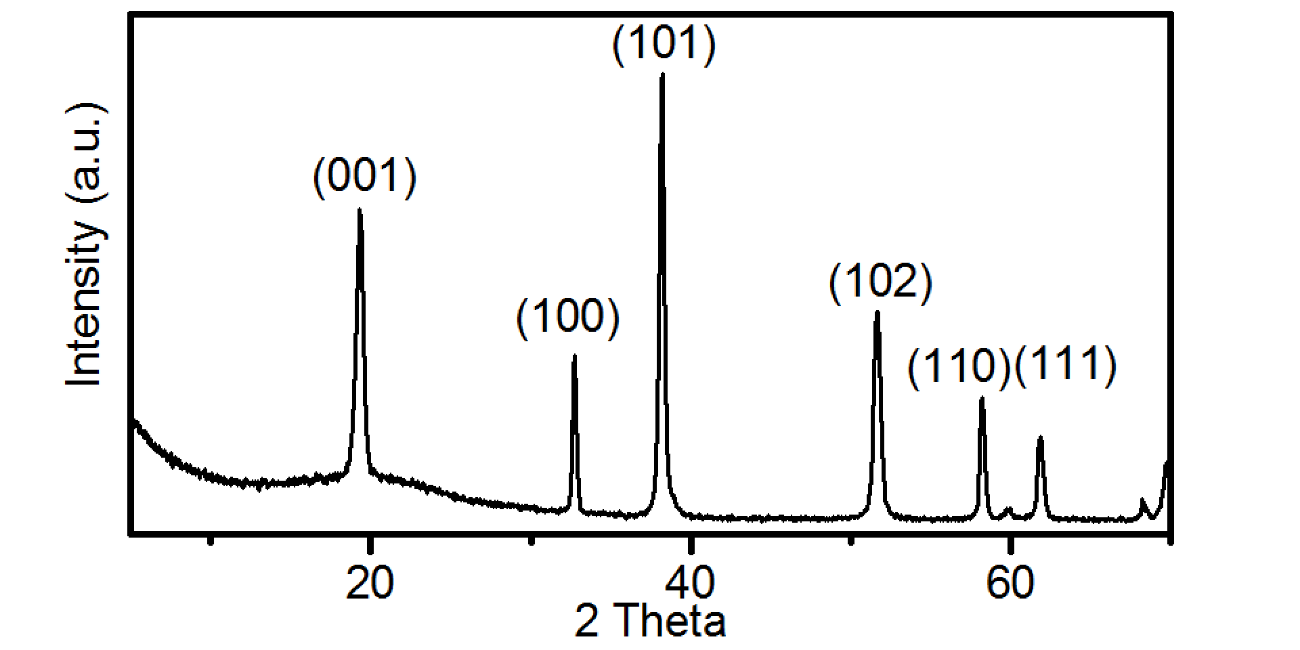
**

**Figure S6** XRD of the prepared Co(OH)_2_ nanosheets.

**
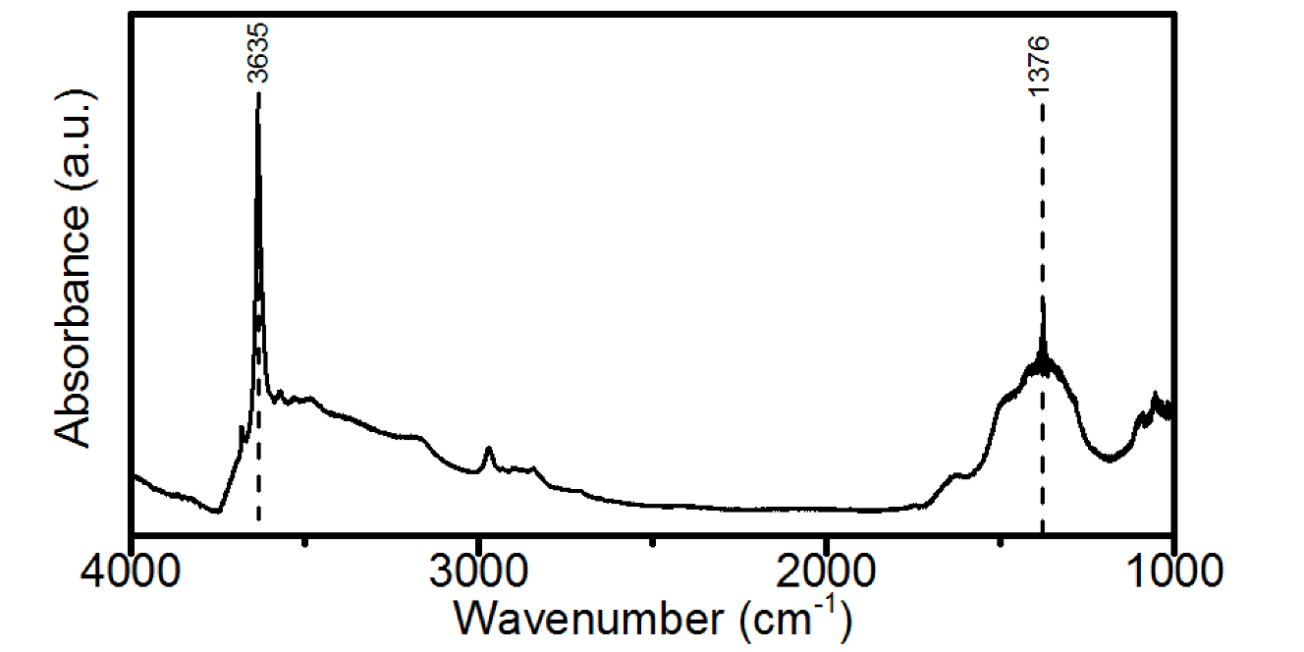
**

**Figure S7** FTIR spectrum of the as-synthesized Co(OH)_2_ nanosheets.

**
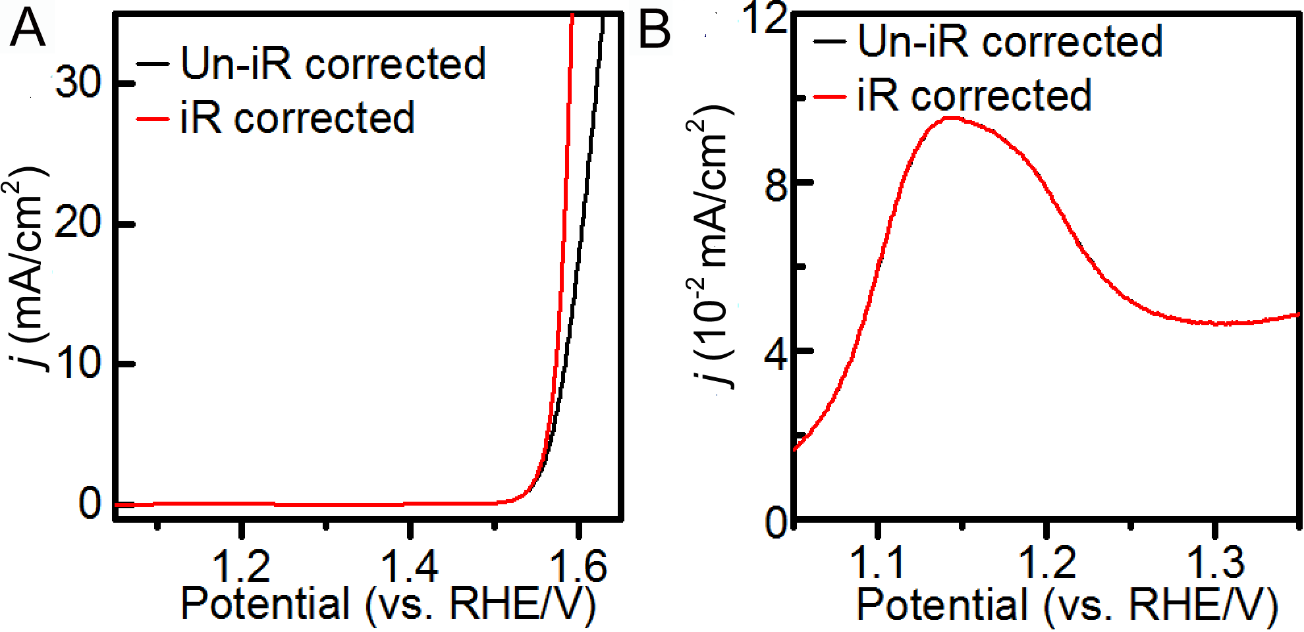
**

**Figure S8** Electrochemical measurements of the Co-MOF nanosheets: iR-corrected (Red) and uncorrected (Black) LSV plots.

**
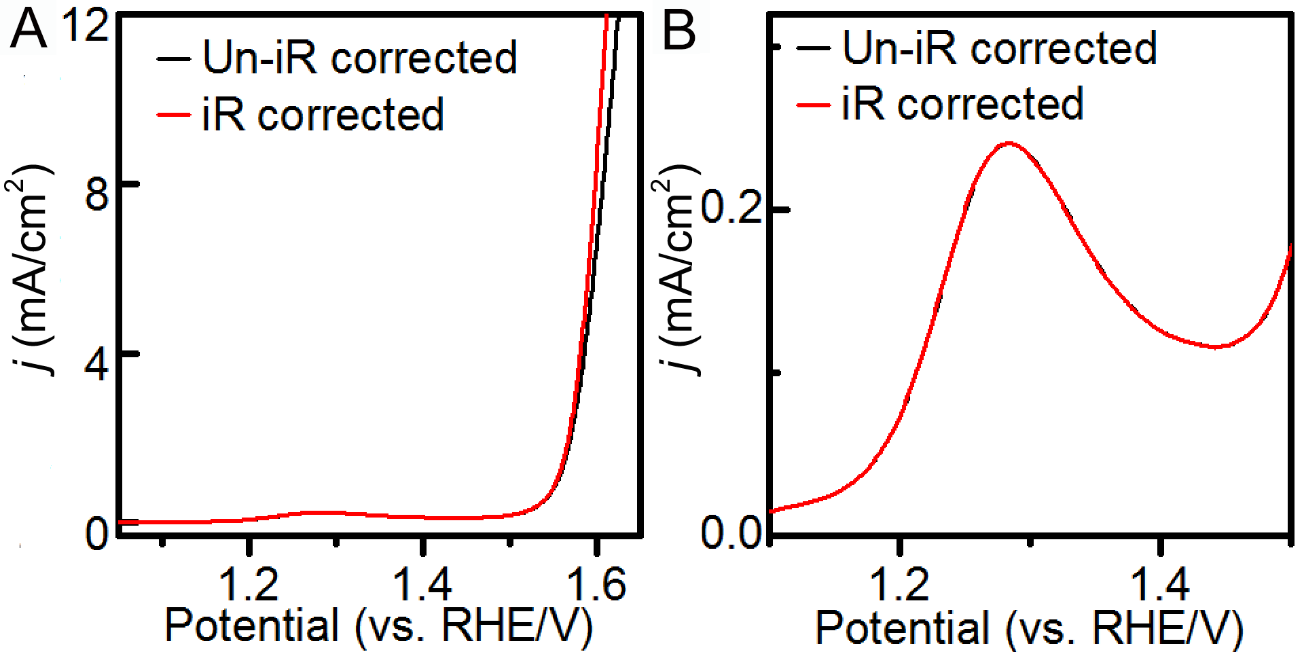
**

**Figure S9** Electrochemical measurements of the bulk MOF-71: iR-corrected (Red) and uncorrected (Black) LSV plots.

**
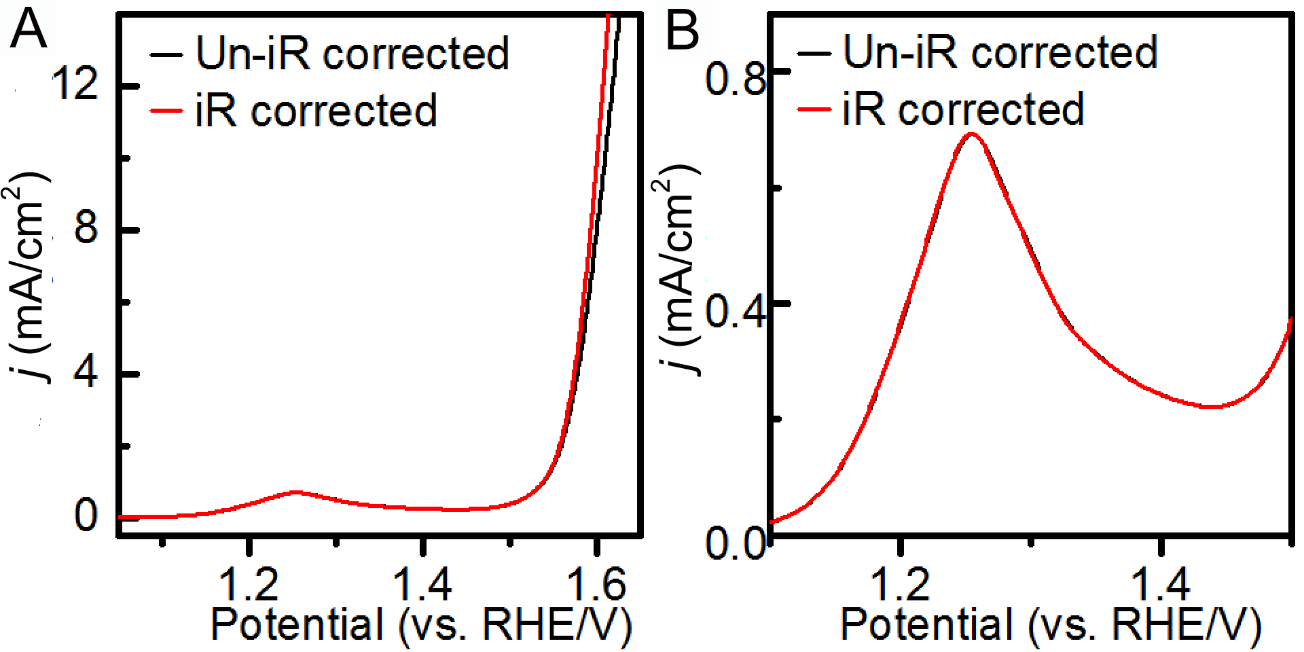
**

**Figure S10** Electrochemical measurements of the Co(OH)_2_ nanosheets: iR-corrected (Red) and uncorrected (Black) LSV plots.

**
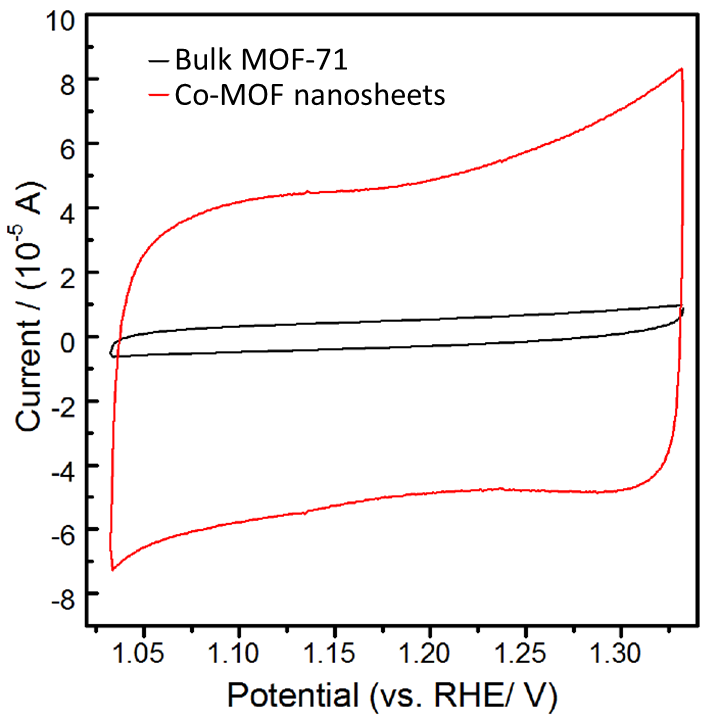
**

**Figure S11** Cyclic voltammetry (CV) measurements of Co-MOF nanosheets (Red) and bulk MOF-71 (Black).

**
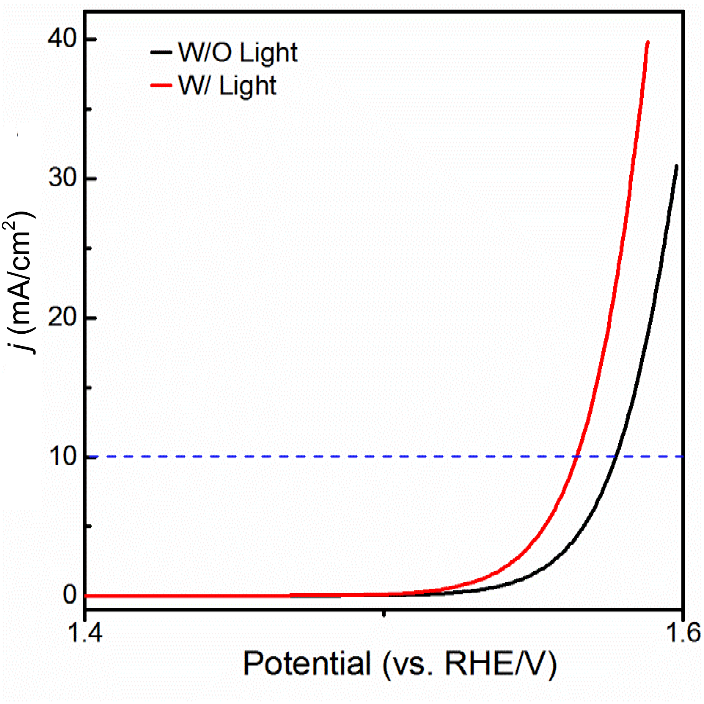
**

**Figure S12** Electrochemical measurements of the Co-MOF nanosheets: iR-corrected LSV plots with and without light illumination.

**
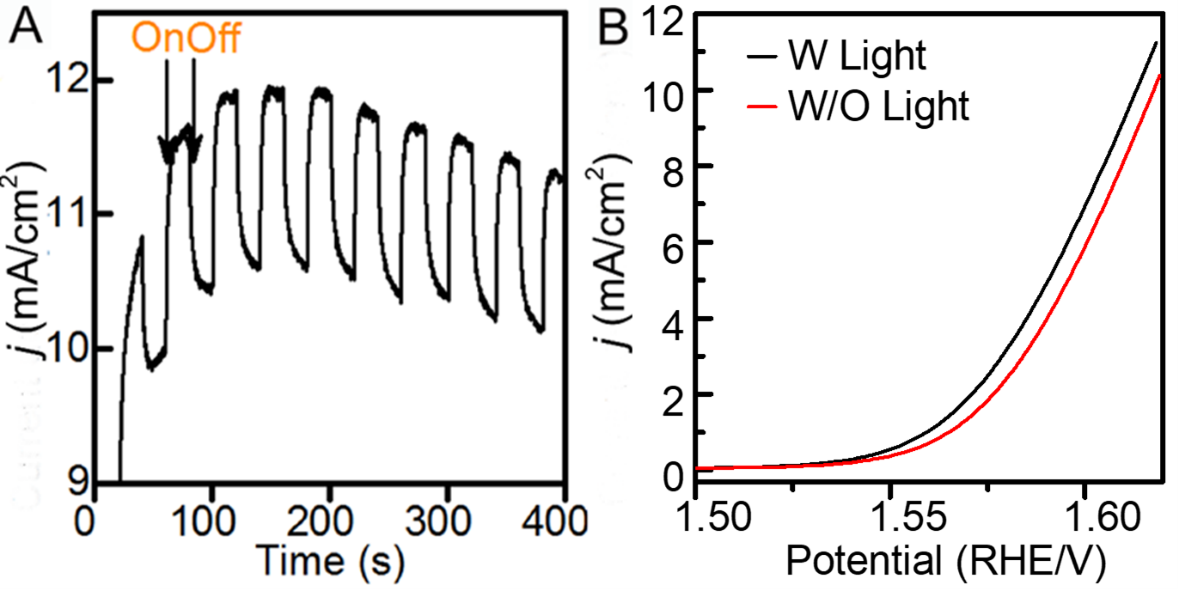
**

**Figure S13** Electrochemical measurements of the bulk MOF-71: **(A)** potentiostatic current response at E= 1.532 V and **(B)** iR-corrected LSV plots with and without light illumination.


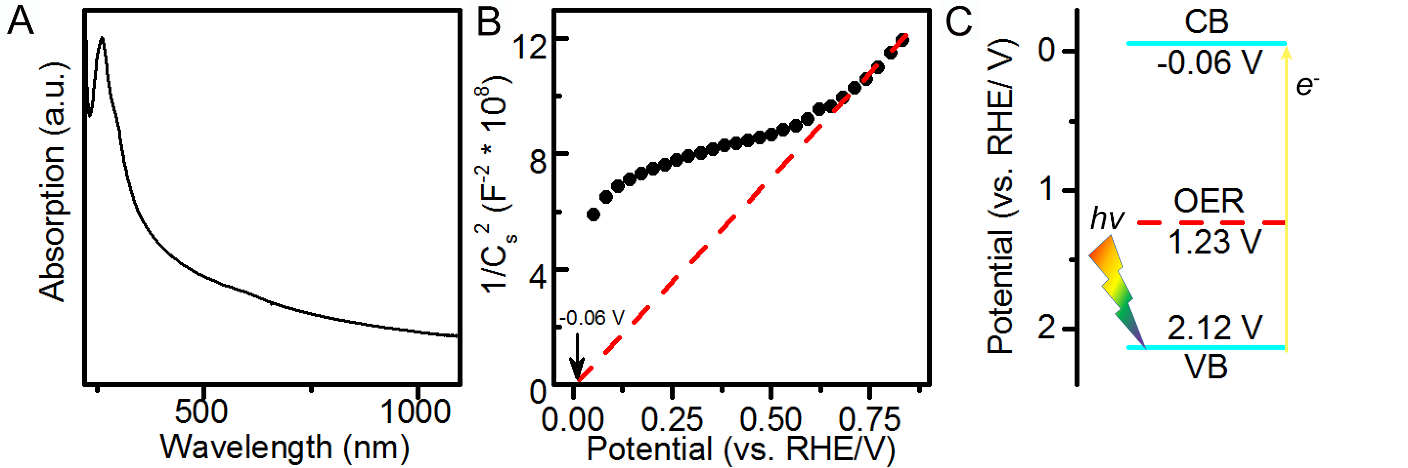


**Figure S14** Characterization of bulk MOF-71, UV-Vis absorption **(A)**, Mott-Schottky plot **(B)**, and Energy band alignment **(C)**.

**
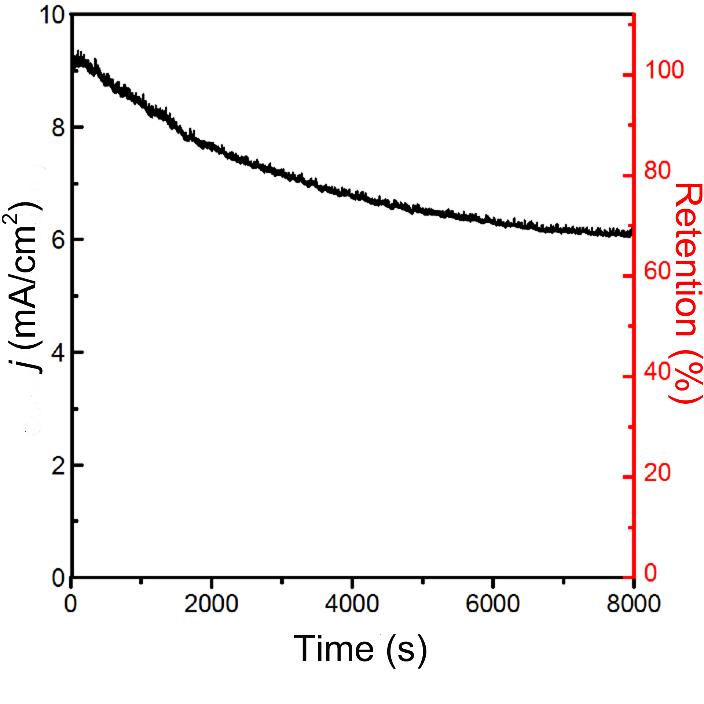
**

**Figure S15** Durability test of the Co-MOF nanosheets at 352 mV overpotential: current density and current density retention as function of time.

**

**

**Figure S16** Electrochemical measurements of the Co-MOF nanosheets: Nyquist plot before and after durability test at 352 mV overpotential.

**
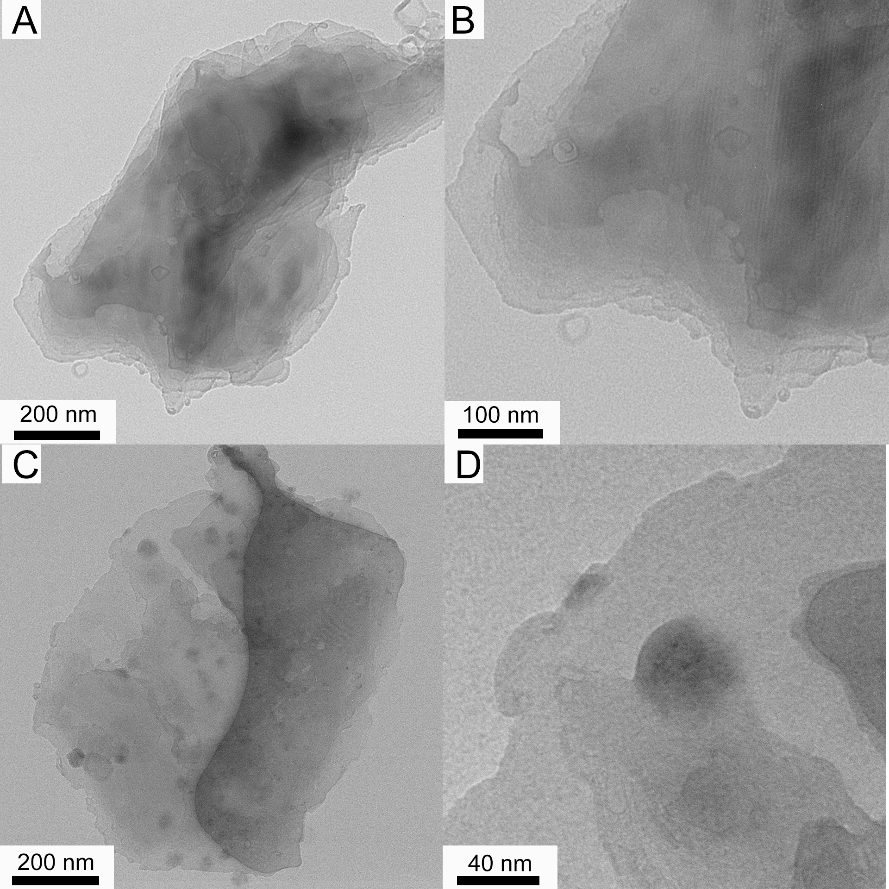
**

**Figure S17** Characterizations of the tested Co-MOF nanosheets: **(A, B)** TEM of the Co-MOF nanosheets after at 302 mV overpotential for 8,000 seconds, and **(C, D)** TEM of the Co-MOF nanosheets after at 352 mV overpotential for 8,000 seconds.

**
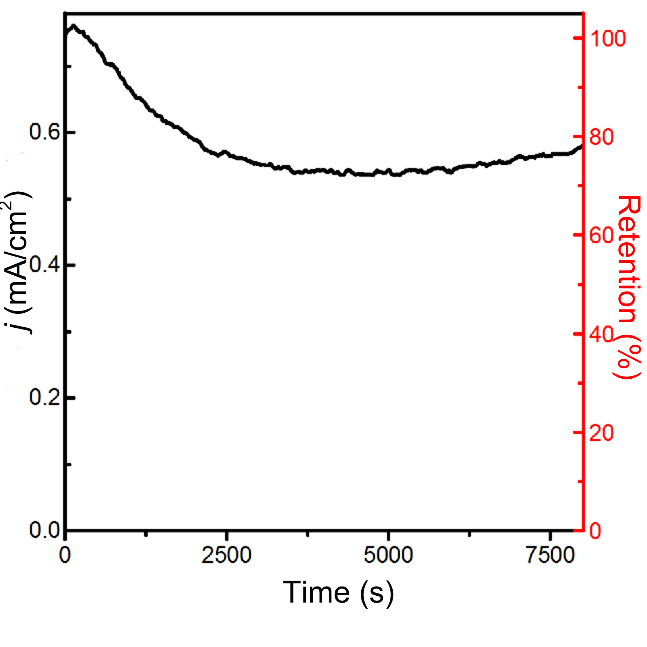
**

**Figure S18** Durability test of the Co-MOF nanosheets at 302 mV overpotential: current density and current density retention as function of time.

.

**
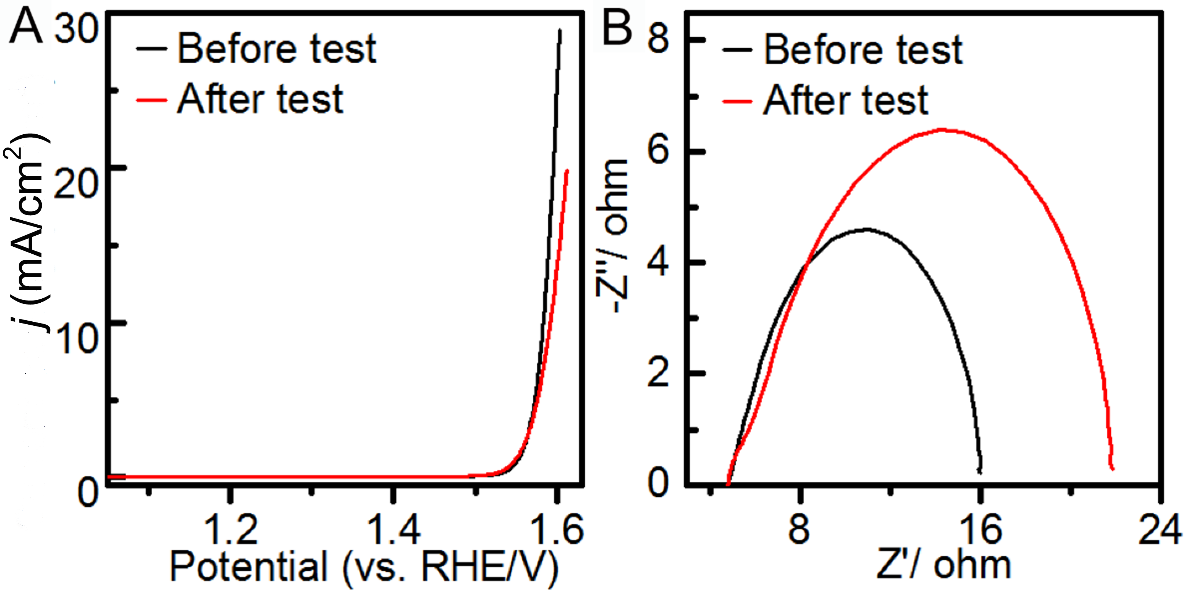
**

**Figure S19** Electrochemical measurements of the Co-MOF nanosheets: **(A)** iR-corrected LSV plots and **(B)** Nyquist plots before and after durability test at overpotential 302 mV.


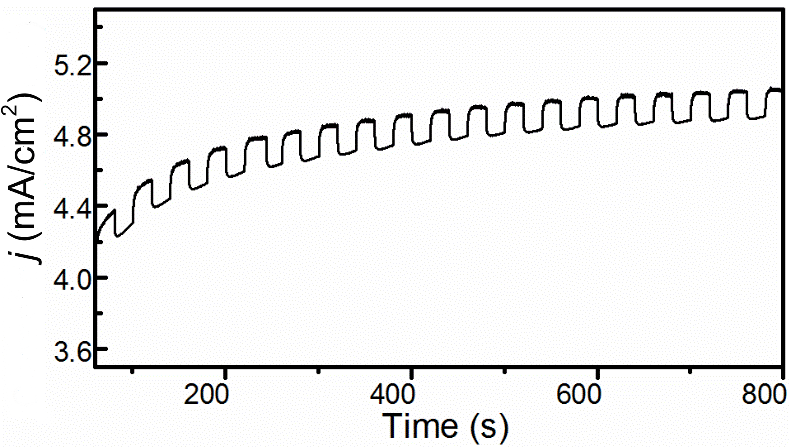


**Figure S20** Electrochemical measurements of the Co-MOF nanosheeets after stability test: potentiostatic current response at E= 1.532 V with and without light illumination.

**Table S1** Summary of OER electrocatalytic activity from recently reported 2D MOF nanosheets and MOF-derived materials.


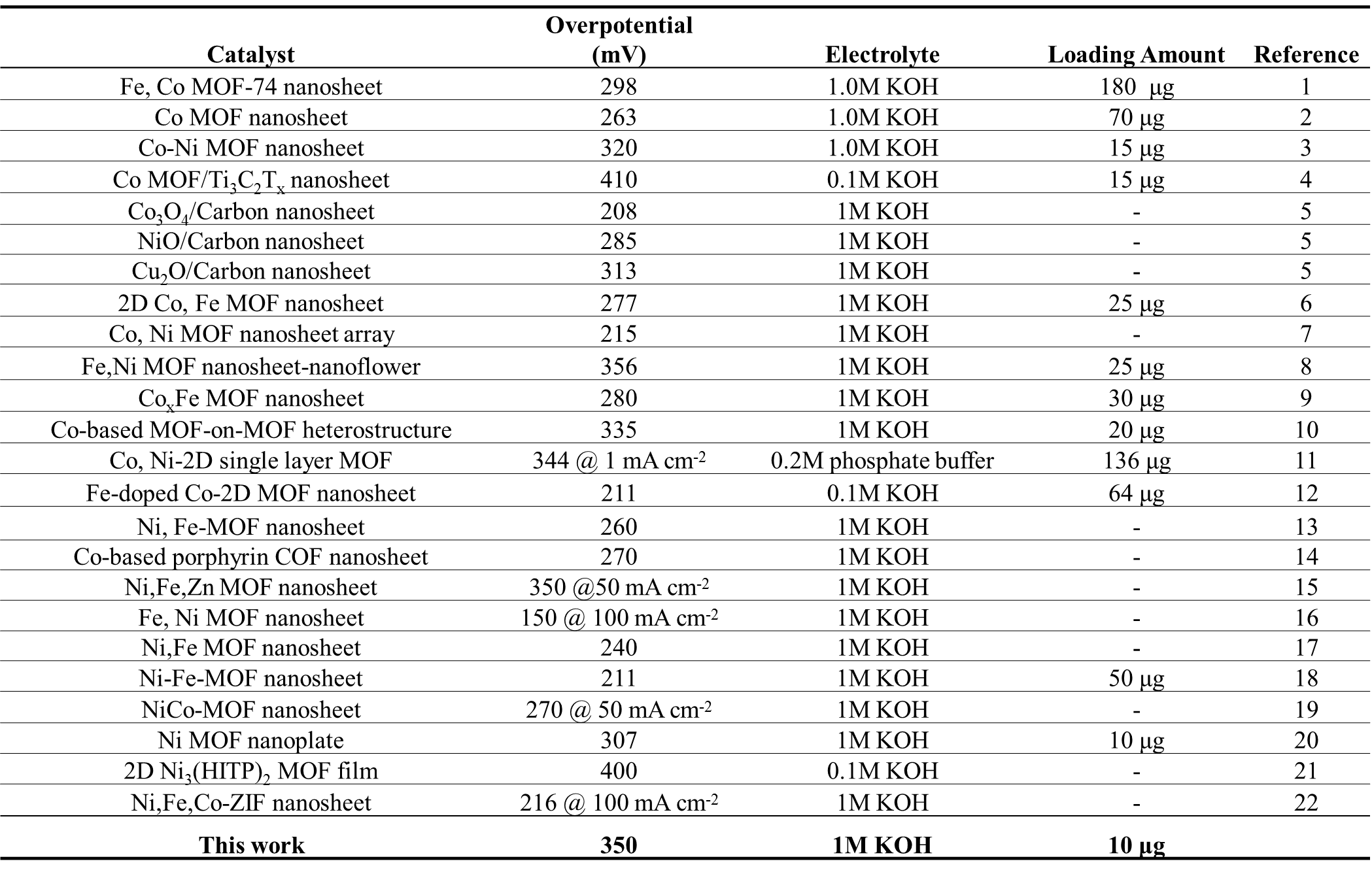


Supplementary References:

1. Zhuang, L., Ge, L., Liu, H., Jiang, Z., Jia, Y., Li, Z., et al. (2019) A surfactant-free and scalable general strategy for synthesizing ultrathin two-dimensional metal–organic framework nanosheets for the oxygen evolution reaction. *Angew. Chem. Int. Ed.* 58, 13565–13572. doi: 10.1002/anie.201907600
2. Xu, Y., Li, B., Zheng, S., Wu, P., Zhan, J., Xue, H., et al. (2018) Ultrathin two-dimensional cobalt–organic framework nanosheets for high-performance electrocatalytic oxygen evolution. *J. Mater. Chem. A* 6, 22070–22076. doi: 10.1039/c8ta03128b
3. Lu, M., Li, Y., He, P., Cong, J., Chen, D., Wang, J., et al. (2019) Bimetallic metal-organic framework nanosheets as efficient electrocatalysts for oxygen evolution reaction. *J. Solid State Chem*. 272, 32–37. doi: 10.1016/j.jssc.2019.01.023
4. Zhao, L., Dong, B., Li, S., Zhou, L., Lai, L., Wang, Z., et al. (2017) Interdiffusion reaction-assisted hybridization of two-dimensional metal−organic frameworks and Ti_3_C_2_T_x_ nanosheets for electrocatalytic oxygen evolution. *ACS Nano* 11, 5800−5807. doi: 10.1021/acsnano.7b01409
5. Zhou, J., Dou, Y., Zhou, A., Shu, L., Chen, Y. and Li, J. R. (2018) Layered metal−organic framework-derived metal oxide/carbon nanosheet arrays for catalyzing the oxygen evolution reaction. *ACS Energy Lett.* 3, 1655−1661 doi: 10.1021/acsenergylett.8b00809
6. Xu, J., Zhu, X. and Jia X. (2019) From low- to high-crystallinity bimetal−organic framework nanosheet with highly exposed boundaries: an efficient and stable electrocatalyst for oxygen evolution reaction. *ACS Sustainable Chem. Eng.* 7, 16629−16639. doi: 10.1021/acssuschemeng.9b03952
7. Huang, L., Gao, G., Zhang, H., Chen, J., Fang, Y., and Dong, S. (2020) Self-dissociation-assembly of ultrathin metal-organic framework nanosheet arrays for effcient oxygen evolution. *Nano Energy* 68, 104296. doi:10.1016/j.nanoen.2019.104296
8. Li, Y., Lu, M., He, P., Wu, Y., Wang, J., Chen, D., et al. (2019) Bimetallic metal-organic framework-derived nanosheet assembled nanoflower electrocatalysts for efficient oxygen

evolution reaction. *Chem. Asian J.* 14, 1590 – 1594. doi: 10.1002/asia.201900328

1. Li, W., Fang, W., Wu, C., Dinh, K. N., Ren, H., Zhao, L., et al. (2020) Bimetal–MOF nanosheets as efficient bifunctional electrocatalysts for oxygen evolution and nitrogen reduction reaction. *J. Mater. Chem. A* 8, 3658–3666. doi: 10.1039/c9ta13473e
2. Zha, Q., Yuan, F., Qin, G. and Ni, Y. (2020) Cobalt-based MOF-on-MOF two-dimensional heterojunction nanostructures for enhanced oxygen evolution reaction electrocatalytic activity. *Inorg. Chem.* 59, 1295−1305. doi: 10.1021/acs.inorgchem.9b03011
3. Pang, W., Shao, B., Tan, X. Q., Tang, Cong, Zhang, Z. and Huang J. Exfoliation of metal–organic frameworks into efficient single-layer metal–organic nanosheet electrocatalysts by the synergistic action of host–guest interactions and sonication. *Nanoscale* 12, 3623–3629. doi: 10.1039/c9nr09742b
4. Huang, J., Li, Y., Huang, R. K., He, C. T., Gong, L., Hu, Q., et al. (2018) Electrochemical exfoliation of pillared-layer metal–organic framework to boost the oxygen evolution reaction. *Angew. Chem.* 130, 4722 –4726. doi: 10.1002/anie.201801029
5. Hai, G., Jia, X., Zhang, K., Liu, X., Wu, Z., and Wang, G. (2017) High-performance oxygen evolution catalyst using two-dimensional ultrathin metal-organic frameworks nanosheets. *Nano Energy* 44, 345–352. doi: 10.1016/j.nanoen.2017.11.071
6. Huang, H., Li, F., Zhang, Y. and Chen, Y. (2019) Two-dimensional graphdiyne analogue Cocoordinated porphyrin covalent organic framework nanosheets as a stable electrocatalyst for the oxygen evolution reaction. *J. Mater. Chem. A* 7, 5575–5582. doi: 10.1039/c9ta00040b
7. Wei, X., Li, N. and Liu, N. (2019) Ultrathin NiFeZn-MOF nanosheets containing few metal oxide nanoparticles grown on nickel foam for efficient oxygen evolution reaction of electrocatalytic water splitting. *Electrochimica Acta* 318, 957-965. doi: 10.1016/j.electacta.2019.06.141
8. Luo, S., Gu, R., Shi, P., Fan, J., Xu, Q., Min, Y. (2020) π-π interaction boosts catalytic oxygen evolution by self-supporting metal-organic frameworks. *J. of Power Sources* 448 227406. doi: 10.1016/j.jpowsour.2019.227406
9. Duan, J., Chen, S., and Zhao, C. (2017) Ultrathin metal-organic framework array for efficient electrocatalytic water splitting. *Nat Commun* 8, 15341. doi: 10.1038/ncomms15341
10. Li, F. L., Wang, P., Huang, X., Young, D. J., Wang, H. F., Braunstein, P., et al. (2019) Large-scale, bottom-up synthesis of binary metal–organic framework nanosheets for efficient water oxidation. *Angew. Chem.* 131, 7125 –7130. doi: 10.1002/anie.201902588
11. Thangasamy, P., Shanmuganathan S. and Subramanian V. (2020) A NiCo-MOF nanosheet array based electrocatalyst for the oxygen evolution reaction. *Nanoscale Adv.* 2, 2073–2079. doi: 10.1039/d0na00112k
12. Lin, Y., Wan, H., Wu, D., Chen, G., Zhang, N., Liu, X., et al. (2020) Metal−organic framework hexagonal nanoplates: bottom-up synthesis, topotactic transformation, and efficient oxygen evolution reaction *J. Am. Chem. Soc.* 142, 7317−7321. doi:10.1021/jacs.0c01916
13. Liu, X. H., Yang, Y. W., Liu, X. M., Hao, Q., Wang, L. M., Sun, B., et al. (2020) Confined synthesis of oriented two-dimensional Ni_3_(hexaiminotriphenylene)_2_ films for electrocatalytic oxygen evolution reaction. *Langmuir* 36, 7528−7532. doi: 10.1021/acs.langmuir.0c01128
14. Ding, M., Chen, J., Jiang, M., Zhang, X. and Wang, G. (2019) Ultrathin trimetallic metal–organic framework nanosheets for highly efficient oxygen evolution reaction. *J. Mater. Chem. A,* 7, 14163-14168. doi: 10.1039/C9TA00708C
